# BDNF Val66Met influences the mediation of sex differences in VLM scores by plasma BDNF in a cohort enriched with risk for Alzheimer‘s disease

**DOI:** 10.1002/alz.088986

**Published:** 2025-01-09

**Authors:** Kyle J Edmunds, Gabriella M Mamlouk, Sarah R Lose, Sanjay Asthana, Mark A. Sager, Matthew Stremlau, Sterling C. Johnson, Henriette van Praag, Ozioma C Okonkwo

**Affiliations:** ^1^ Wisconsin Alzheimer's Disease Research Center, School of Medicine and Public Health, University of Wisconsin‐Madison, Madison, WI USA; ^2^ Geriatric Research Education and Clinical Center William S. Middleton VA Hospital, Madison, WI USA; ^3^ Wisconsin Alzheimer's Disease Research Center, University of Wisconsin School of Medicine and Public Health, Madison, WI USA; ^4^ Wisconsin Alzheimer's Institute, University of Wisconsin School of Medicine and Public Health, Madison, WI USA; ^5^ Lab of Neurosciences, National Institute on Aging (NIA), Baltimore, MD USA; ^6^ Geriatric Research Education and Clinical Center, William S. Middleton Memorial Veterans Hospital, Madison, WI USA; ^7^ Wisconsin Alzheimer's Disease Research Center, Madison, WI USA; ^8^ Wisconsin Alzheimer’s Institute, University of Wisconsin‐Madison School of Medicine and Public Health, Madison, WI USA; ^9^ Florida Atlantic University, Jupiter, FL USA; ^10^ University of Wisconsin, Madison, WI USA

## Abstract

**Background:**

Brain‐derived neurotrophic factor (BDNF)—a key neurotrophin involved in synaptic plasticity, neurogenesis, and neuroprotection—has been shown to mediate sex differences in verbal learning and memory (VLM) ability, but it remains unclear whether this relationship is conditionally dependent upon carriage of the Val66Met polymorphism in the BDNF gene. This study investigates how BDNF_Val66Met_ carriage influences the mediation of sex differences in VLM scores by plasma BDNF levels in a cohort enriched for AD risk.

**Method:**

Cognitively unimpaired participants in the Wisconsin Registry for Alzheimer’s Prevention (WRAP; n=198, age 63.8±6y, 66% women, 66% family history of AD, 38% apolipoprotein E4 (APOE‐ε4) carriers, 31% BDNF_Val66Met_ carriers) underwent the Rey Auditory Verbal Learning Test (RAVLT). Scores from learning trials 3‐5 and the delayed recall test were aggregated as a VLM performance index. Plasma BDNF levels were measured using a Human BDNF Quantikine Immunoassay (R&D Systems). Striatified mediation analysis and bootstrapping were performed to test the conditional dependence of BDNF mediation on BDNF_Val66Met_ carriage, and model covariates included age, APOE‐ε4 carriage, parental history of AD, education, hippocampal volume, and date difference between VLM and plasma data acquisition.

**Result:**

Stratified mediation models showed a significant association between sex and VLM scores in BDNF_Val66Met_ carriers [β=‐0.61; p=0.04], but no significant association between sex and BDNF levels [β=0.06; p=0.85]. By comparison, in BDNF_Val66Val_ homozygotes, women had significantly higher BDNF levels [β=‐0.62; p<0.01] and VLM scores [β=‐0.77; p<0.01], and bootstrapping showed BDNF to be a significant partial mediator of the effect of sex on VLM [β=‐0.14; 95% CI: ‐.292, ‐.021].

**Conclusion:**

This study indicates that BDNF_Val66Met_ carriage may attenuate the mediating role of plasma BDNF expression on the relationship between sex and VLM scores.

